# Case report: Self-performed orthopedic exams in telehealth treatment of a youth athlete with acute rotator cuff strain

**DOI:** 10.3389/fspor.2023.1150850

**Published:** 2023-05-30

**Authors:** Yuxuan Wang, Nuo Yi, Hayley M. Ericksen, Wupeng Zhang

**Affiliations:** ^1^ PT Solutions, Portland, OR, United States; ^2^ University of Oklahoma Health Sciences Center, Tulsa, OK, United States; ^3^ University of Wisconsin-Milwaukee, Milwaukee, WI, United States; ^4^ Nankai University School of Medicine, Tianjin, China

**Keywords:** telehealth, sports rehabilitation, orthopedic examination, rotator cuff strain, youth athletes

## Abstract

**Background:**

Sports-related rotator cuff muscle injury is one of the most prevalent pathologies affecting overhead sports athletes. Since the COVID-19 pandemic and its subsequent stay-at-home protocols, physical therapy has transited into a new realm of telehealth. Current evidence regarding examining and managing RTC strain in telehealth physical therapy is minimal.

**Case presentation:**

A self-referred 14-year-old female Chinese semi-professional tennis player presented with an acute right RTC strain. The mechanism of injury involved forehand strokes with left trunk rotation. No ligamentous or labral damage was observed on Magnetic Resonance Imagining. The individualized care plan included virtual partner-assisted assessment, online instructions on therapeutic exercises, and education with psychosocial considerations.

**Outcome and follow-up:**

After a 6-week intervention, the patient demonstrated complete shoulder range of motion, full muscle strength, complete return-to-practice, 0% Quick DASH disability index, and 6/68 on the Tampa Scale for kinesiophobia.

**Discussion:**

This case report demonstrated that telehealth is an accessible and cost-effective option for youth tennis athletes with RTC strain. This unique case showed a detailed roadmap from examination to discharge of this plan of care. There are also barriers including test and measure validity, and communication difficulties to be considered. Despite the challenges, this case was a good example of telehealth being an effective, repeatable, and cost-efficient option for patients with poor healthcare access.

## 1. Introduction

### 1.1. Background

Since the COVID-19 pandemic and its subsequent stay-at-home protocols, physical therapy (PT) has transitioned into telehealth (TH). The American Physical Therapy Association defined TH as “the use of electronic information and telecommunication technologies to remotely provide healthcare information and services” (1). With its increasing usage in orthopedic rehabilitation, TH provides several benefits including greater access, convenience, reduced cost, and improved patient self-efficacy by promoting high-quality communication, thorough history, individualized education, and shared-decision-making (2–5). With its superior accessibility, it would be beneficial to investigate TH's effectiveness in high-prevalence musculoskeletal injuries such as sports-related rotator cuff (RTC) muscle injuries among overhead-sports athletes (6, 7). Current evidence indicated the effectiveness of in-person PT in managing RTC-related pain, however, no high-level evidence study has described TH effectiveness (8–10). Despite its many benefits, TH also poses several challenges, including HIPAA compliance, patient preference for hands-on interventions, and particularly, difficulties in performing tests and measures (5, 11).

Overall, the development of TH allows PT to break geographical restrictions. Despite its current progression, an effective and reliable method to measure the strength and special tests from TH requires further exploration. The purpose of this case report is to describe the successful management of an acute RTC injury in a youth semi-professional tennis player through telehealth. CARE guideline (for CAse REports) was followed when preparing this manuscript.

### 1.2. Case presentation

The patient was a 14-year-old female Chinese semi-professional tennis athlete. She first developed mild right shoulder pain after a forehand tennis stroke with left trunk rotation in practice which disappeared after two days of resting. The second injury occurred five days later with the same mechanism of injury and resulted in 8/10 pain with immediate loss of upper extremity function. An x-ray and Magnetic Resonance Images (MRI) of the patient's shoulder were ordered by her primary care physician. The imagines ruled out epiphyseal plate, ligamentous, or labral damage, but showed a supraspinatus muscle strain. The patient self-referred to our team for TH due to the limited access to local sports-medicine resources. Oral consent was obtained from her guardian. During TH history taking, the patient complained of weakness with shoulder flexion, abduction, and external rotation (ER), as well as kinesiophobia. These combined physical and psychological limitations negatively impacted the patient's activities of daily living (ADL) and tennis. (Details in Table 1).

**Table 1:** Case presentations.

Case presentations	
Age	14
Gender	Female
Initial Patient Reported Outcome Measure	Quick DASH 57.5% Sports and Performance Arts Module 75% Tampa Scale for Kinesiophobia: 48/68
Initial NPRS score at worst (0–10)	8
Initial Special Tests	Painful Arc (+) painful Drop Arm (-) painful Infraspinatus test (+) painful Hawkins-Kennedy (+)painful
Radiographic Findings	MRI: no labral or ligamentous pathologies identified

WFL, within functional limits; deg, degree; NPRS, Numerical pain rating scale; MRI, magnetic resonance imaging.

**Figure 1:**
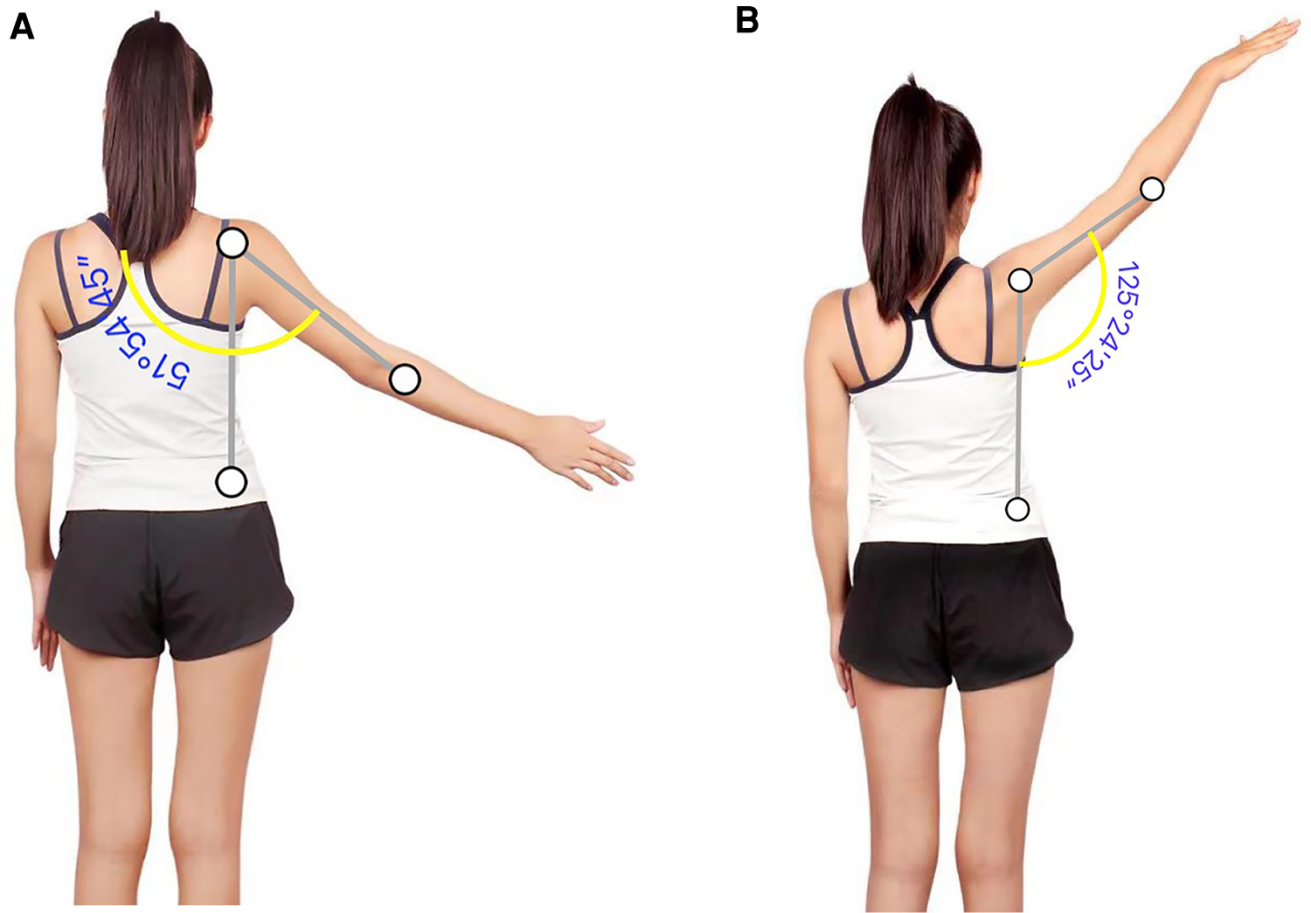
(**A,B**) R shoulder abduction of 2-dimensional analysis in pre-intervention and 1-week post-intervention.

After collecting patient history, the clinicians conducted a 30-minute virtual training session with the patient's guardian to teach hand placements, resistance strength, force angle for manual muscle testing (MMT), and special tests. For range of motion (ROM) measurements, the clinicians screenshot the patient's shoulder active ROM (AROM), then measured the angles through a smartphone 2-Dimensional application (Angle Meter 360) after the assessment (12) (Figure 1). For strength tests, modified MMT (partner-assisted MMT) was used. Instead of having the tester grade on a 0 to 5 scale, the patient reported a percentage of effort by answering the question: “Compared to your uninvolved side, how many percent were you able to resist on the last repetition?” Special tests cluster (Painful Arc, Drop-arm test, Infraspinatus test, and Hawkins-Kennedy) were performed by the guardian and showed positive signs (13, 14). (Detailed testing results can be found in Table 1).

### 1.3. Differential diagnosis

Based on patient demographic, history, inspection, and ROM testing, the clinicians highly suspected a strain/minor tear of one or more RTC muscles, with less likely diagnoses of subacromial impingement, deltoid strain, and acromioclavicular joint sprain. Since the quality of tests for specific RTC muscles is lacking, the test cluster of Painful Arc, Drop-arm, and Infraspinatus test was used, which has a 91% probability of RTC tear if all three are positive (14). With two out of the three tests being positive, a positive Hawkins-Kennedy test, and images, it is reasonable to diagnose the patient with RTC strain and general subacromial pain syndrome. Stage-1 primary impingement could also be supported by the results; however, this wouldn't change the management strategy. The patient has an excellent prognosis in conservative treatment based on her age, absence of previous medical history, and absence of epiphyseal plate injury.

### 1.4. Therapeutic interventions

Telehealth re-evaluation and treatments were delivered once per week, over 6 weeks. Each re-evaluation session included 10 min of subjective report related to overall exercises intensity, pain level, 10–15 min of re-assessment, and 15 min for patient education including home-exercise-program technique instructions, intensity tapering using rate of perceived exertion scale (RPE), activity modification and precautions, and psycho-social management. The patient's guardian assisted with the re-evaluation of ROM, and MMT in the same manner as the initial evaluation to ensure reliability. Between visits, the patient recorded herself performing the home exercises program (HEP) and sent videos to therapists weekly. The clinicians provided asynchronous education and feedback in the form of video and writing, which focused on form correction, RPE monitoring and tapering, and patient encouragement, within 12 h of receiving the recordings. The asynchronous nature was practically unavoidable due to time-zone differences, where the patient was located in China and the clinicians in different parts of the US.

**Figure 2:**
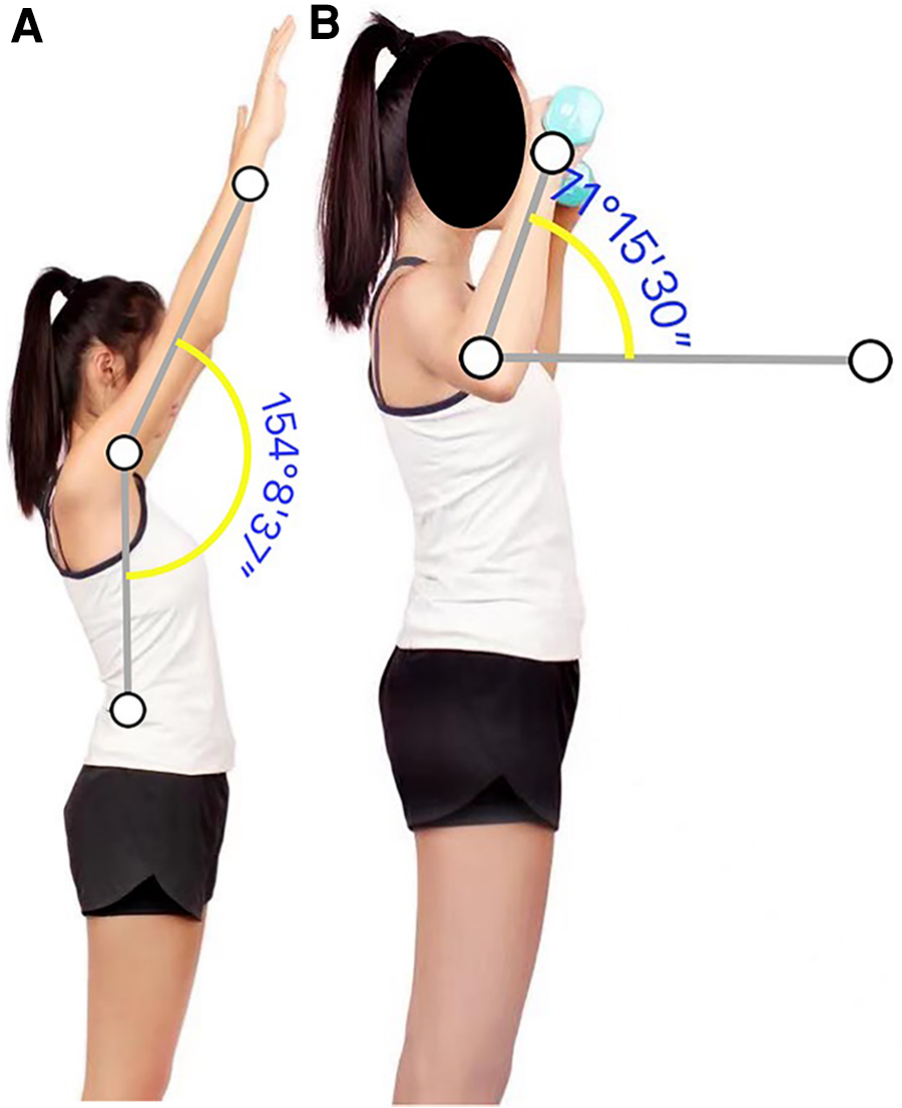
(**A,B**) R shoulder active flexion and resistance exercises with 1 kg dumbbell in the 2nd week of intervention.

The plan of care was divided into three phases. The initial phase (0–2 weeks) aimed to reduce pain, improve shoulder ROM and increase strength of the scapular muscles. Therapeutic exercises included active-assistive-ROM (Figure 2) with low loads (<6/10 RPE) and high repetitions to restore para-scapular muscle movement control, RTC muscle strength, and joint proprioception. The intermittent phase (2–4 weeks) aimed to progressively improve strength of the shoulder and para-scapular muscles by incorporating high resistance (8–9/10 RPE) and low volumes. Exercises in this phase included progressive stretching, close-kinetic-chain proprioception re-training, and eccentric/concentric isotonic exercises. The return-to-sports phase (4–6 weeks) was considered a buffering stage between rehabilitation and full practice participation. This phase included upper extremity plyometrics and graded exposure to tennis-specific motions. Wilk et al's (15) protocol was utilized: 50% to 75% intensity for stroke <180 times and serve <80 times total for the first week of return-to-sports. The intensity gradually increased by approximately 10% each week until 100% (15). Close status monitoring and goal tracking allowed the clinicians to make appropriate decisions on a return-to-sport timeline. (See details of education and interventions in Tables 2, 3).

**Table 2:** Education and modalities.

Educations	Modalities	Duration and frequency
Avoid therapeutic exercises that trigger pain sensation >2/10		
Avoid heavy excessive of Ext, Add and IR or combined motion	Massage	10 min as needed, 3×/wk after therapeutic exercise
Avoid excessive stretching or sudden movement	Therapeutic US (Heat)	15 min, 3×/wk before HEP for the first week at local clinics (US only, no other treatment)
Avoid sleeping on the injured side (R)		
Avoid weight bearing activities on injured site (R)		
Pain free motion and position for daily activity		
Take note of any change or onset of new symptoms		

EXT, extension; IR, internal rotation; ADD, adduction; R, right; min, minute; wk, week.

**Table 3:** Interventions.

Week 1		Week 2–4		Week 4–6		Week 7	
AAROM D1	3 × 15 Dowel	Flexion	3 × 15 2 kg DB	Cat-cow stretch	3 × 15 N/A	Corner stretch	3 × 1 min Wall
Shoulder pendulum	3 × 15 Table	Resisted D1	3 × 15 1 kg band	Corner stretch	3 × 1 min Wall	Standing horizontal stretch	3 × 1 min N/A
AROM Flexion	3 × 15 N/A	Wall corner stretching	3 × 1 min Wall	Standing horizontal stretch	3 × 1 min N/A	50% intensity serving practice	<80× total for the first 3 days
Scapular squeeze	3 × 15 N/A	Side-lying ER/IR	3 × 15 2 kg DB	Sitting IR	3 × 15 2 kg band	75% intensity serving practice	<80× total for the rest of the 1st week
AROM Scaption	3 × 15 N/A	Rhythmic stab	Wall N/A	Standing abduction	3 × 15 2 kg DB	50% ball contact stroke	<180× total for the 1st week
Isometric ER	3 × 15 × 5 s Towel roll	Scaption	3 × 15 1 kg band	Plank on elbows	3 × 45 s N/A		
Isotonic IR	3 × 15 × 5 s 2 kg band	IR	3 × 15 3 kg band	Knee push up	3 × 15 N/A		
Elbow extension	3 × 15 2 kg band	Sleeper's stretch	3 × 1 min N/A	No ball bat swing	3 × 20 Tennis bat		
Serratus punch	3 × 15 N/A	Serratus punch	3 × 15 2 kg DB	No ball serving	3 × 20 Tennis bat		

AROM, active range of motion; AAROM, active assistive range of motion; ER, external rotation; IR, internal rotation; ADD, adduction; DB, dumbbell.

### 1.5. Follow-ups and outcomes

At 2 weeks follow-up, the patient demonstrated full pain-free shoulder ROM and self-reported 70% muscle strength on the modified partner-assisted MMT compared to the healthy side of flexion/abduction and 90% of ER. At week 4, the patient self-reported 100% strength in all shoulder muscles, with fully functional ADL including carrying, lifting, and pulling. Special test cluster showed all negatives when re-tested. After 5 weeks of rehabilitation, the patient started tennis-specific movements such as no-ball contact strokes and serves practice, then gradually returned to play with light-intensity ball contact. Before the patient fully returned to practice, her Quick Disabilities of the Arm, Shoulder, and Hand (DASH) resulted in 0% dysfunction with 0% disability in the Sports module, and 6/68 on Tampa Scale for kinesiophobia (lower is better for each). The patient and her guardian were satisfied with the outcome. One-month follow-up included an interview with the patient and her guardian regarding her current symptoms during tennis practice. The patient stated that her shoulder returned to prior level of function without any limitations. (Detailed initial and discharge outcome measurements in Table 4).

**Table 4:** Initial and final findings.

Evaluation	Initial exam result	2 weeks	4 weeks	6 weeks
Initial AROM	Flexion ∼90 deg (pain) Scaption ∼90 deg (pain) Abduction ∼51 deg (pain) (angles recorded at the first instance of pain, the patient has more AROM with pain) Adduction WFL Extension WFL (pain) External Rotation Limited (pain) Internal Rotation WFL Horizontal Abduction WFL (pain) Shoulder shrug WFL	Flexion - WNL w/o pain Scaption - WNL w/o pain Abduction - WNL w/o pain Adduction - WNL w/o pain Extension - WNL w/o pain External Rotation WNL - w/o pain Internal Rotation WFL - w/o pain Horizontal Abduction WFL w/o pain Shoulder shrug WFL w/o pain	All ROM are as prior levels.	No limitation in all ROM compared to the healthy side. Self-reported 100% muscle strength and negative cluster special tests
Strength Testing (Self-reported by comparing to the uninvolved side)	Flexion 60% at 80 deg (pain) Scaption 60% at 80 deg (pain) Abduction 60% at 90 deg (pain) Extension 100% (mild pain) External Rotation 50% at 30 deg (pain) Internal Rotation 90% (pain) Shoulder shrug WFL	Scaption 70% w/o pain Abduction 70% w/o pain ER 90% w/o pain All others approximately equal compare bilaterally	No pain for all MMT, self-reported MMT approximately 100% Self-reported quick fatigue during tennis-specific drills	Self-reported 100% muscle strength and negative cluster special tests No limitations for tennis practice
Outcome measure	Quick Dash 57.5%, Sports Module 75% Tampa Scale for Kinesiophobia: 48/68	Quick Dash 37.8%, ports module: 60%		Quick Dash 0%, sports module 0% Tampa Scale for Kinesiophobia: 6/68

WFL, within functional limits; deg, degree.

## 2. Discussion

This case report is an excellent example of TH management for an acute sports-related RTC strain leading to desirable outcomes.

A strength of this case is the clinicians’ comprehensive approach to treatment despite delivering care in a 100% virtual environment. The lack of physical contact allowed the clinicians to engage the patient in self-assessment of her status and strengthen therapeutic alliance; both are significant positive prognostic factors for a successful recovery. Another strength of this report is the detailed roadmap of a novel remote examination approach and treatment, including a return-to-sports protocol that is appropriate for a TH program. This provides necessary resources for future TH providers when managing a motivated injured youth athlete.

There are several weaknesses in this TH case report. These challenges include the ability to objectively assess injury status, treatments with asynchronous feedback, and the nature of TH communication. The first challenge is measuring muscle strength and ensuring the validity of special tests. Previous case reports utilized AAROM, self-resisted MMT, and self-administered special tests, however, none included a volumetric measurement (7, 16). In this case for ROM measurement, two-dimensional analysis was used and has previously been shown to have good to excellent validity and intra-rater reliability (17). For strength, the clinicians chose to modify MMT to allow patient self-report to be part of the process. This encouraged the patient to assess internally and further engage in rehabilitation. This case showed that the partner-assisted MMT/orthopedic special test combined with a self-reported scale could be a viable tool in TH.

Another challenge is the significant amount of effort the clinicians put in to educate the guardian on MMT/special test techniques. The author recognized that this 30 min training session may not be feasible for every practice setting. There is no current research on gold standards for MMT, ROM, and special test measurements in TH, and future investigations are recommended. However, the authors still recommend TH providers give clear instructions for self or partner-assisted testing before each test/re-test to maximize the precision and validity of tests and measures. Although the clinicians in this case had the benefit of imaging to support diagnosis, taking a comprehensive patient history remains a heavily weighted diagnostic tool in TH (18).

Our final challenge lies in asynchronous feedback that potentially impacted outcomes negatively. Though previous research had shown that instruction using booklets and videos was effective in the long term (6 m, 1 yr, 2 yr timepoints), education such as form correction, or intensity tapering became less efficient and may discourage patient participation (11). This negative effect was minimized by focusing patient education on the importance of following exercise instructions and emphasizing the use of RPE during HEP. The outcome was also favorable due to the patient's high motivation to return to sports. The driver of success in this case further reinforced the importance in TH of establishing therapeutic alliances and promoting patient self-efficacy. Lastly, the patient indicated that this was the only way she could receive the level of care without traveling and lodging in another city, which would have incurred higher costs and interrupted school. This case is a good example of how TH PT could be an effective and more accessible option for patients in underserved areas.

Despite the limitations presented in this case, it demonstrated that TH is an achievable, cost-efficient, and repeatable procedure for delivering physical therapy care. Future TH studies should focus on exploring the validity of the examining process and objective testing.

## 3. Conclusion

In conclusion, this unique international telehealth case study demonstrated a successful episode of remote rehabilitation for a high-level teenage athlete with RTC muscle strain. This case is also a pioneering example of exploring the potential of TH in PT evaluations and treatments and improving patients’ accessibility to PT in underserved populations.

## 4. Patient perspective

The patient was contacted again one month following discharge. She reported that she had returned fully to tennis practice and continued with her independent HEP to maintain shoulder health.

## Data Availability

The original contributions presented in the study are included in the article, further inquiries can be directed to the corresponding author.

## References

[1] American Physical Therapy Association. Telehealth modalities PTs and PTAs can use during the public health emergency.

[2] Bradford N Caffery L Smith A. Correction: telehealth services in rural and remote Australia: a systematic review of models of care and factors influencing success and sustainability. Rural Remote Health. (2016) 16(4):1–24. 10.22605/RRH4268 27817199

[3] Guo Y Albright D. The effectiveness of telehealth on self-management for older adults with a chronic condition: a comprehensive narrative review of the literature. J Telemed Telecare. (2018) 24(6):392–403. 10.1177/1357633X17706285 28449619

[4] Powell RE Henstenburg JM Cooper G Hollander JE Rising KL. Patient perceptions of telehealth primary care video visits. AnnFam Med. (2017) 15(3):225–9. 10.1370/afm.2095 PMC5422083 28483887

[5] Turner A. Case studies in physical therapy: transitioning a “hands-on” approach into a virtual platform. Int J Telerehabil. (2018) 10(1):37. 10.5195/ijt.2018.6253 30147842 PMC6095681

[6] Pluim BM Staal JB Windler GE Jayanthi N. Tennis injuries: occurrence, aetiology, and prevention. Br J Sports Med. (2006) 40(5):415–23. 10.1136/bjsm.2005.023184 16632572 PMC2577485

[7] Zeligson E Donlon L Camarinos J. Treatment of hip and knee pain, with a focus on return to running, using telemedicine: 2 case reports. JOSPT Cases. (2022) 2(1):13–7. 10.2519/josptcases.2022.10191

[8] Alrabaa RG Lobao MH Levine WN. Rotator cuff injuries in tennis players. Curr Rev Musculoskelet Med. (2020) 13(6):734–47. 10.1007/s12178-020-09675-3 32827301 PMC7661672

[9] Hopewell S Keene DJ Heine P Marian IR Dritsaki M Cureton L Progressive exercise compared with best-practice advice, with or without corticosteroid injection, for rotator cuff disorders: the GRASP factorial RCT. Health Technol Assess (Rockv). (2021) 25(48):1–58. 10.1016/S0140-6736(21)00846-1 PMC9421560 34382931

[10] Longo UG Risi Ambrogioni L Berton A Candela V Carnevale A Schena E Physical therapy and precision rehabilitation in shoulder rotator cuff disease. Int Orthop. (2020) 44(5):893–903. 10.1007/s00264-020-04511-2 32157371

[11] Hall JL McGraw D. For telehealth to succeed, privacy and security risks must be identified and addressed. Health Aff. (2014) 33(2):216–21. 10.1377/hlthaff.2013.0997 24493763

[12] Cunha AB Babik I Harbourne R Cochran NJ Stankus J Szucs K Assessing the validity and reliability of a new video goniometer app for measuring joint angles in adults and children. Arch Phys Med Rehabil. (2020) 101(2):275–82. 10.1016/j.apmr.2019.07.008 31465759

[13] Michener LA Doukas WC Murphy KP Walsworth MK. Diagnostic accuracy of history and physical examination of superior labrum anterior-posterior lesions. J Athl Train. (2011) 46(4):343–8. 10.4085/1062-6050-46.4.343 21944065 PMC3419145

[14] Park HB Yokota A Gill HS El Rassi G McFarland EG. Diagnostic accuracy of clinical tests for the different degrees of subacromial impingement syndrome. JBJS. (2005) 87(7):1446–55. 10.2106/JBJS.D.02335 15995110

[15] Wilk KE Bagwell MS Davies GJ Arrigo CA. Return to sport participation criteria following shoulder injury: a clinical commentary. Int J Sports Phys Ther. (2020) 15(4):624. 10.26603/ijspt20200624 33354395 PMC7735686

[16] Young SW Young TW MacDonald CW. Physical therapist identification of an undetected rotator cuff tear via a telehealth evaluation: a case report. JOSPT Cases. (2021) 1(1):29–33. 10.2519/josptcases.2021.9990

[17] Lade H McKenzie S Steele L Russell TG. Validity and reliability of the assessment and diagnosis of musculoskeletal elbow disorders using telerehabilitation. J Telemed Telecare. (2012) 18(7):413–8. 10.1258/jtt.2012.120501 23086982

[18] Hampton JR Harrison MJ Mitchell JR Prichard JS Seymour C. Relative contributions of history-taking, physical examination, and laboratory investigation to diagnosis and management of medical outpatients. Br Med J. (1975) 2(5969):486–9. 10.1136/bmj.2.5969.486 1148666 PMC1673456

